# Supplementary material for: Towards a unifying phylogenomic framework for tailed phages
Source: PLoS Genet. 2025 Feb 5;21(2):e1011595. doi: 10.1371/journal.pgen.1011595 (PMC11835377; doi:10.1371/journal.pgen.1011595)
Supplement: S1 Text — Fig A. Phylogenies of INPHARED genomes with at least 5 hits to the 0.5% VOG subset (a) or 0.25% VOG subset (b). Branch colors and outer ring color correspond to ICTV family. Trees are both rooted at the midpoint. Fig B. Phylogeny of all Caudo genomes examined that contained at least 5 hits to the 0.5% VOG subset (11,621 Caudo genomes). Branch colors and outer ring color correspond to ICTV family. Nodes with 200–500 members were collapsed for visualization. (a) phylogeny with genome QGNH01001383.1 of the peat metagenomic dataset excluded as its branch length was exceedingly long and shrunk the visibility of the other leaves and their family designations. (b) phylogeny with QGNH01001383.1 included. Fig C. Representative Caudo 0.5% VOG subset phylogeny (3,052 genomes), with branches colored by family, yellow dots in outer ring corresponding to the presence of the repressor cI VOG (VOG01128) and purple dots to the presence of the integrase VOG (VOG00020). Fig D. DNA polymerase A phylogeny. Branches and outer strip colors correspond to family. Fig E. inCaudo phylogeny of Fig 1B with order designated in outer ring. Table A. Number of VOGs found in a given percent of Caudo genomes and tree quality values from phylogenetic reconstruction with inCaudo genomes. (DOC) [file pgen.1011595.s004.doc]

**S1 Text.**

**Supplemental Figures:**


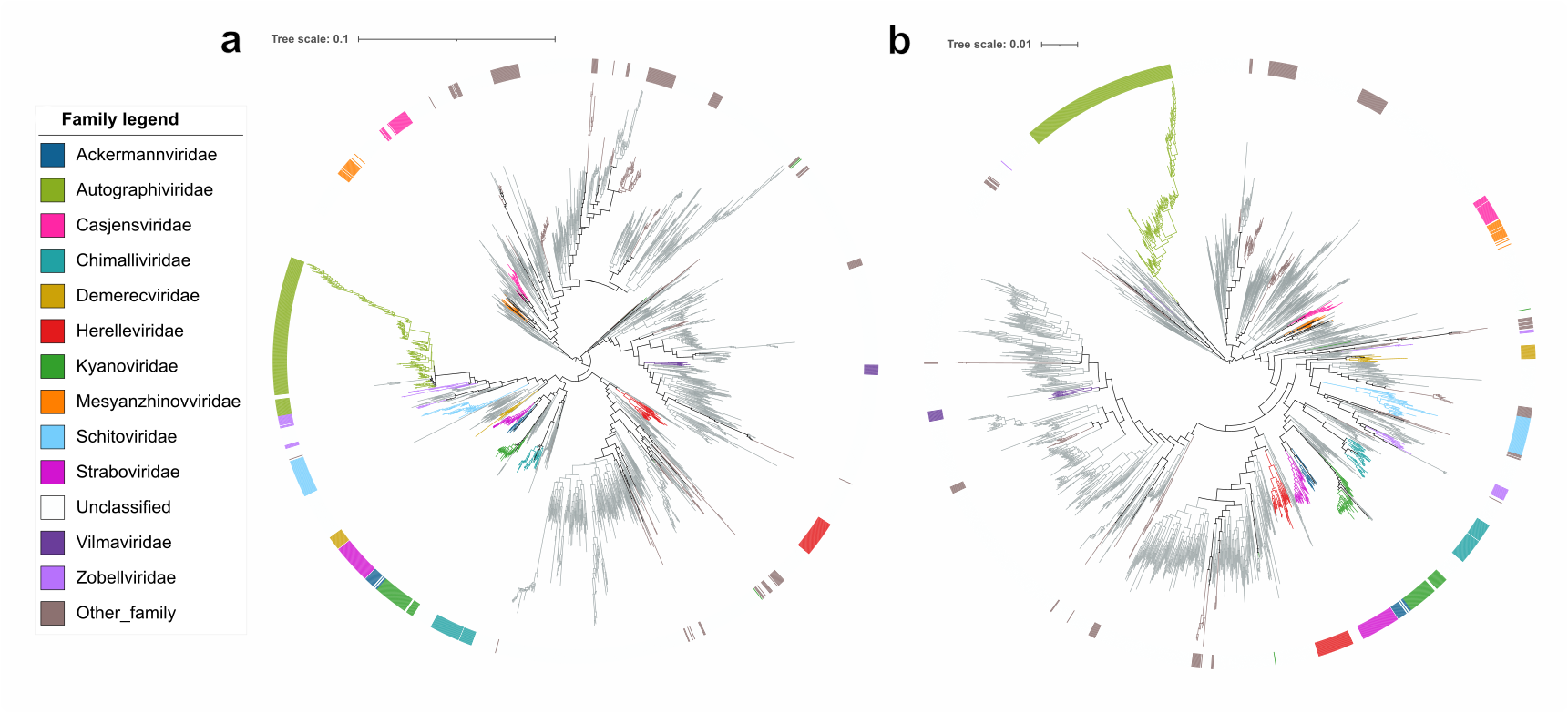


**Fig A.** Phylogenies of INPHARED genomes with at least 5 hits to the 0.5% VOG subset (**a**) or 0.25% VOG subset **(b**). Branch colors and outer ring color correspond to ICTV family. Trees are both rooted at the midpoint.


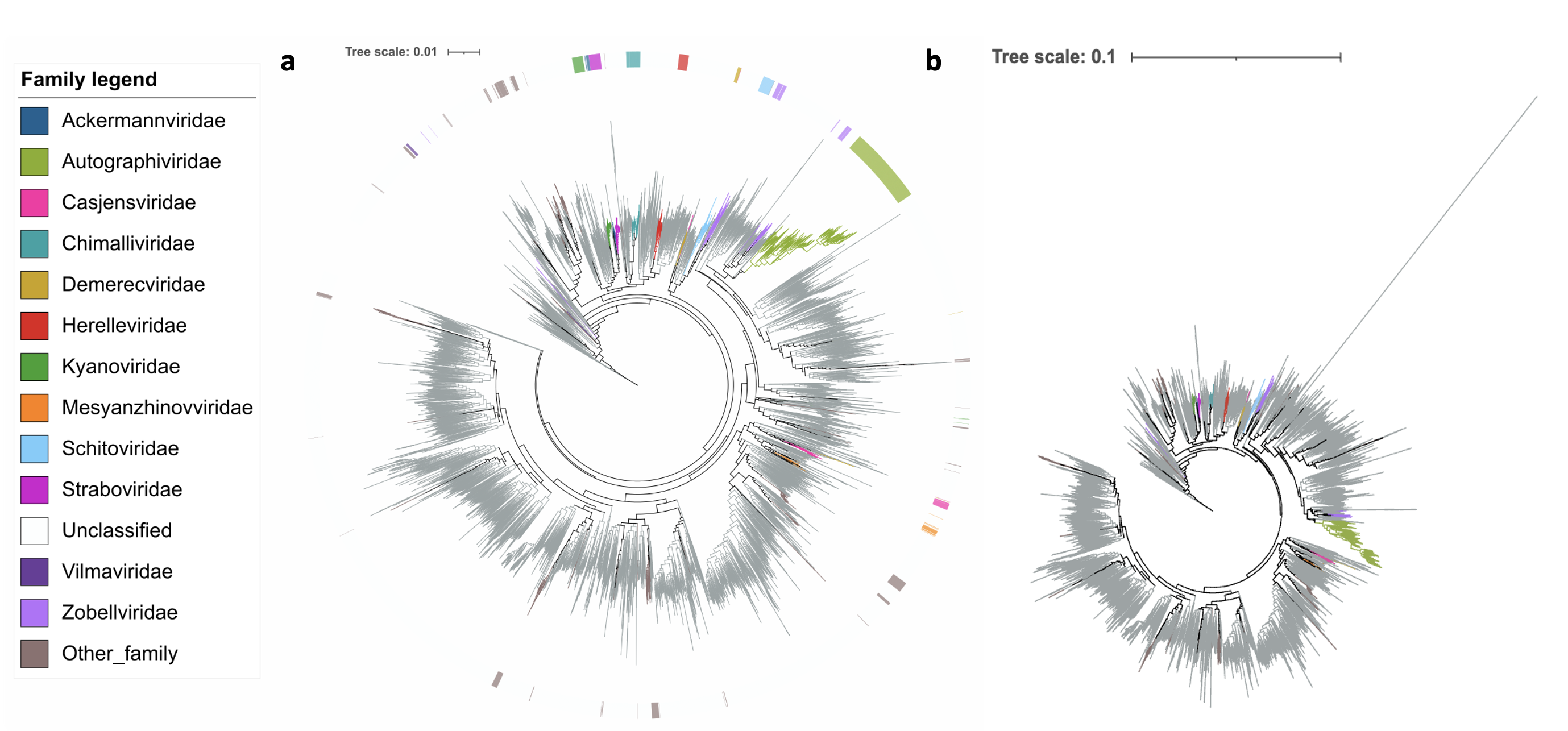


**Fig B**. Phylogeny of all Caudo genomes examined that contained at least 5 hits to the 0.5% VOG subset (11,621 Caudo genomes). Branch colors and outer ring color correspond to ICTV family. Nodes with 200-500 members were collapsed for visualization. (**a**) phylogeny with genome QGNH01001383.1 of the peat metagenomic dataset excluded as its branch length was exceedingly long and shrunk the visibility of the other leaves and their family designations. (**b**) phylogeny with QGNH01001383.1 included.


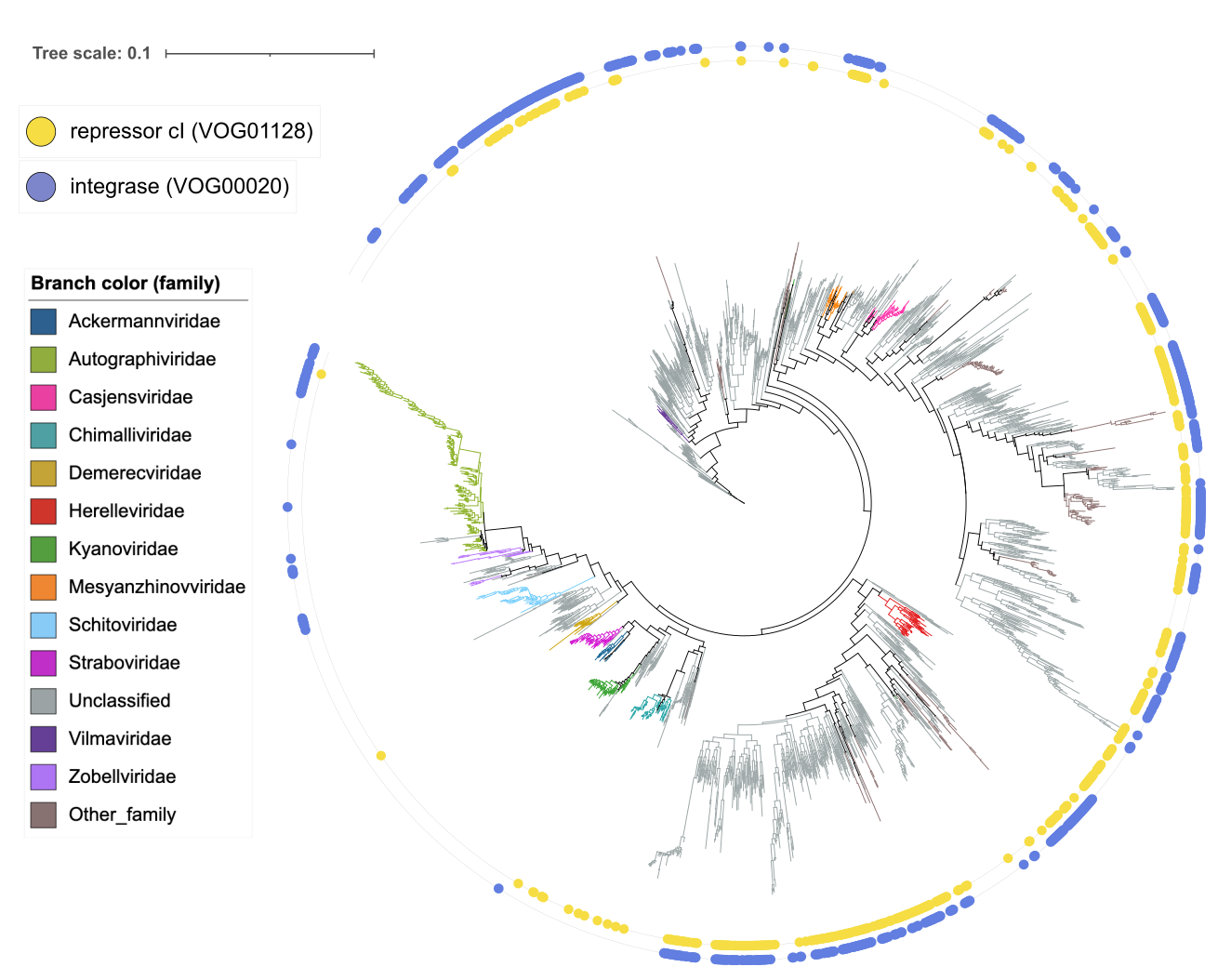


**Fig C.** Representative Caudo 0.5% VOG subset phylogeny (3,052 genomes), with branches colored by family, yellow dots in outer ring corresponding to the presence of the repressor cI VOG (VOG01128) and purple dots to the presence of the integrase VOG (VOG00020).


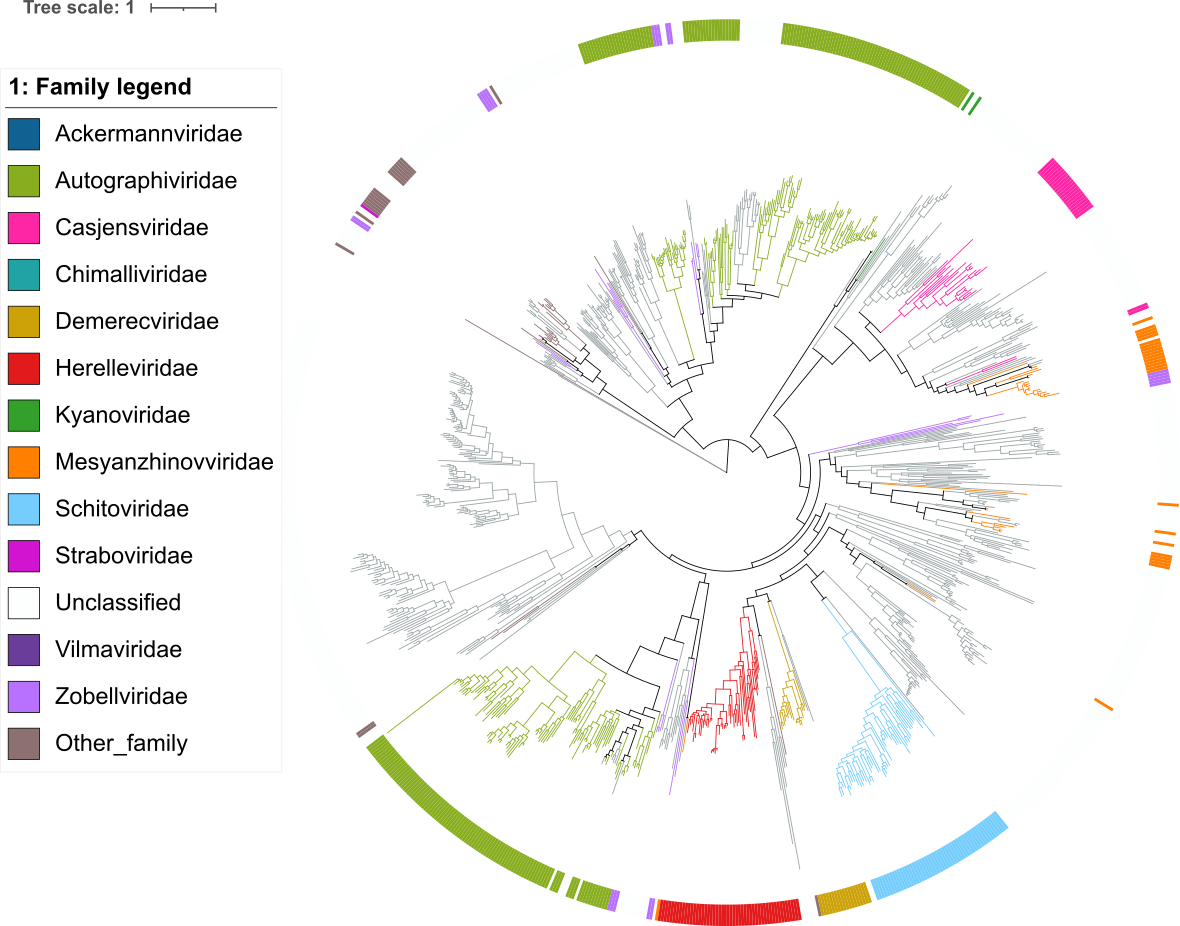


**Fig D.** DNA polymerase A phylogeny. Branches and outer strip colors correspond to family.


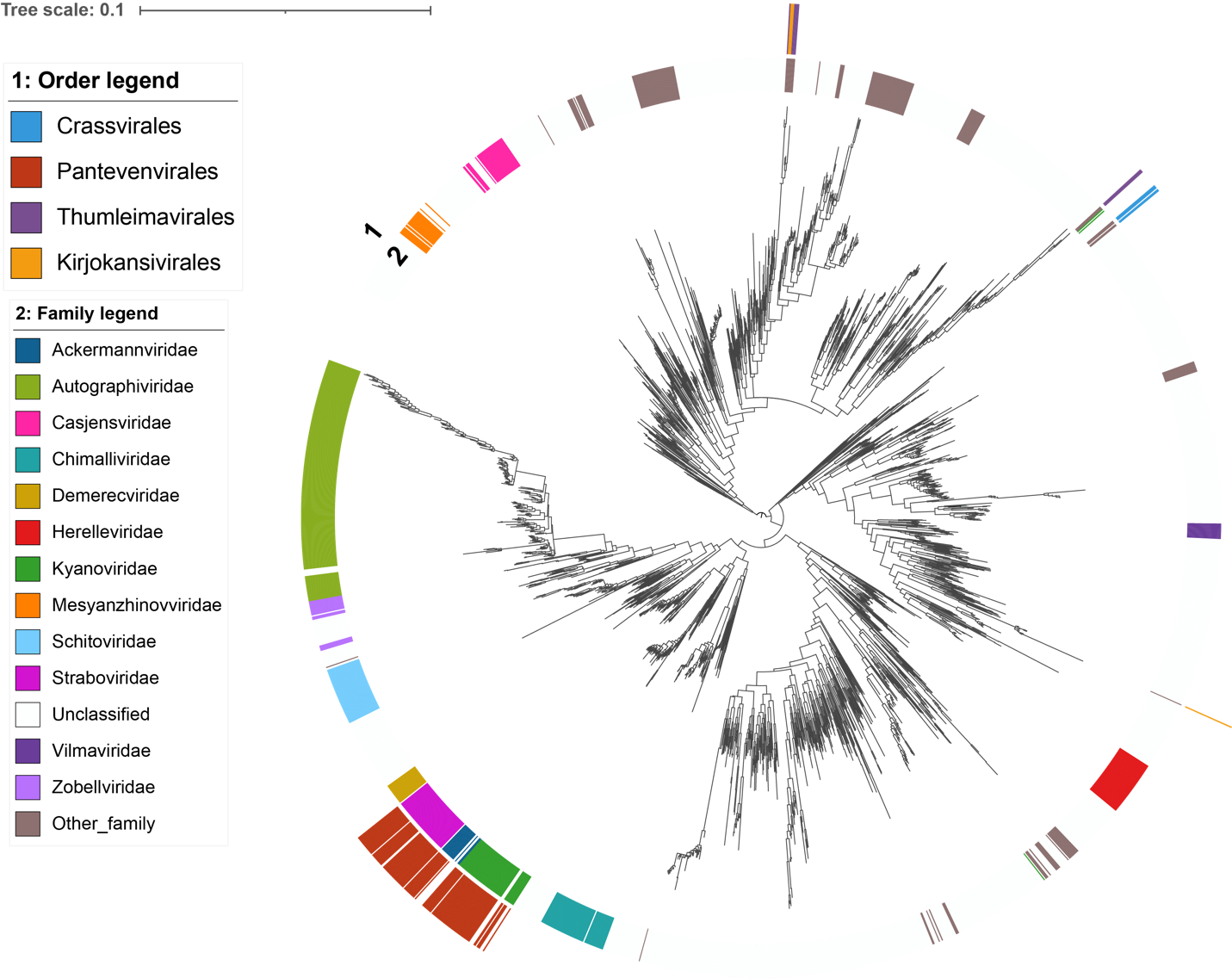


**Fig E.** inCaudo phylogeny of Fig 1b with order designated in outer ring.

**Supplemental Tables**

**Table A.**  Number of VOGs found in a given percent of Caudo genomes and tree quality values from phylogenetic reconstruction with inCaudo genomes.

| Percent of genomes | Number of VOGs | Abbrev | Tree Certainty | Relative Tree  Certainty (0-1) |
| --- | --- | --- | --- | --- |
| 0.25 | 4042 | 025p | 1621.83298 | 0.641548 |
| 0.5 | 2114 | 05p | 1630.17211 | 0.644847 |
| 1 | 1032 | 1p | 1545.86242 | 0.614902 |
| 2 | 472 | 2p | 1292.08113 | 0.513138 |
